# Cognitive Rehabilitation for Neurocognitive Late Effects in Adult Survivors of Childhood Acute Lymphoblastic Leukemia: A Feasibility and Case-Series Study

**DOI:** 10.3389/fpsyg.2021.724960

**Published:** 2021-10-28

**Authors:** Kaja Solland Egset, Siri Weider, Jan Stubberud, Odin Hjemdal, Ellen Ruud, Magnus Aassved Hjort, Mary-Elizabeth Bradley Eilertsen, Anne Mari Sund, Magnhild Eitrem Røkke, Trude Reinfjell

**Affiliations:** ^1^Department of Psychology, Norwegian University of Science and Technology, Trondheim, Norway; ^2^Department of Psychology, University of Oslo, Oslo, Norway; ^3^Department of Research, Lovisenberg Diakonale Hospital, Oslo, Norway; ^4^Department of Pediatric Medicine, Oslo University Hospital, Oslo, Norway; ^5^Faculty of Medicine, University of Oslo, Oslo, Norway; ^6^Department of Clinical and Molecular Medicine, Norwegian University of Science and Technology, Trondheim, Norway; ^7^Children’s Clinic, St. Olavs Hospital, Trondheim University Hospital, Trondheim, Norway; ^8^Department of Public Health and Nursing, Center for Health Promotion Research, Norwegian University of Science and Technology, Trondheim, Norway; ^9^Regional Centre for Child and Youth Mental Health and Child Welfare, Norwegian University of Science and Technology, Trondheim, Norway; ^10^Kavli Institute for Systems Neuroscience, Norwegian University of Science and Technology, Trondheim, Norway

**Keywords:** acute lymphoblastic leukemia, childhood cancer, late effects, rehabilitation, executive functions, adult survivor

## Abstract

Neurocognitive late effects following the diagnosis and treatment of childhood acute lymphoblastic leukemia (ALL) commonly include impaired executive functions (EFs), with negative consequences for one’s health-related quality of life and mental health. However, interventions for EF impairments are scarce. Thus, the aims of this study were to (1) explore the feasibility and acceptability of the cognitive rehabilitation program Goal Management Training (GMT) and (2) examine whether GMT is associated with positive outcomes across cases of ALL survivors with EF complaints. Four participants (median age 31.5 years) underwent nine GMT modules in a total of five group sessions. Rehabilitation was focused on compensatory strategies to improve real-life EFs. Participants were evaluated at 2-week and 6-month follow-ups. Evaluation of feasibility and acceptability included adherence, a semi structured interview, self-reports, and safety. Additionally, therapists’ experience with implementation was evaluated. Outcome measures included self-reports and neurocognitive tests of attention, EF, and processing speed. All participants completed GMT and rated the intervention as useful, suitable, and satisfactory. The reliable change index showed improved daily life EFs (two participants) and neurocognitive performance (three participants) at 6-month follow-up. Additionally, all participants improved on a “real-life” task involving EF. A future randomized controlled trial is recommended.

## Introduction

Since the 1970s, prognosis has progressively improved for childhood acute lymphoblastic leukemia (ALL), and 5-year survival is approaching 90% (Smith et al., 2014; Toft et al., 2018; Jeha et al., 2019). However, despite the optimization of chemotherapeutic agents used in modern ALL treatment protocols (Siegel et al., 2019), the administration of multiple drugs, including dexamethasone and methotrexate, still carries a risk of long-term neurocognitive late effects (Krull et al., 2013b; Cheung and Krull, 2015; Phillips et al., 2020).

Neurocognitive late effects following childhood ALL diagnosis and treatment with either chemotherapy only (CTO) or cranial radiation therapy (CRT) have now been widely documented (Campbell et al., 2007; Janzen and Spiegler, 2008; Castellino et al., 2014; Cheung and Krull, 2015; Hutchinson et al., 2017). Although less severe than those seen in survivors of childhood brain tumors (Krull et al., 2018), ALL survivors show significant late effects in several neurocognitive functions. These most commonly include problems with attention (Conklin et al., 2012; Jacola et al., 2016), processing speed (Edelstein et al., 2011; Kanellopoulos et al., 2016) and executive functions (EFs) (Walsh et al., 2015; Liu et al., 2018). EFs comprise higher-level abilities such as planning, decision-making, and effective performance (Lezak et al., 2012). EFs are dependent on lower level executive abilities such as set-shifting, updating of working memory and inhibition (Miyake et al., 2000; Diamond, 2013). Importantly, EFs are needed to maintain and manage goals (Friedman and Miyake, 2017) and are thereby highly relevant to daily life functioning. Unfortunately, the widely distributed and intricate nature of EF brain sites (Lezak et al., 2012) makes them particularly susceptible to injury.

Several risk factors for neurocognitive late effects, including a higher treatment burden, such as intravenous/intrathecal methotrexate, higher chemotherapy doses or CRT (Hardy et al., 2018; Krull et al., 2018), genetic dispositions (Krull et al., 2008, 2013a), younger age at diagnosis (Edelstein et al., 2011; Kesler et al., 2018) and female sex (Kadan-Lottick et al., 2010; Krull et al., 2013b; Jacola et al., 2016), have emerged. Unfortunately, studies on long-term survivors suggest that neurocognitive late effects may persist (and even increase) well into adulthood (Krull et al., 2013b, 2018; Kanellopoulos et al., 2016). The increasing expectations and demands that accompany adulthood appear to make adult survivors of childhood ALL particularly vulnerable to adverse outcomes (e.g., unemployment) that may follow EF impairments (Mody et al., 2008; Kunin-Batson et al., 2011; Krull et al., 2013b). Moreover, they report lower health-related quality of life (HRQoL) and poorer mental health status than age-matched controls (Kanellopoulos et al., 2013). However, there is a gap in the literature regarding cognitive rehabilitation of adult survivors of childhood ALL, and there is currently no EF rehabilitation available for this group of survivors.

One of the most extensively studied cognitive rehabilitation programs is Goal Management Training (GMT) (Levine et al., 2011). GMT is a structured and group-based program originally developed for subjects with acquired brain injury (ABI) (Levine et al., 2000). The program is based on cognitive behavioral principles, with a special focus on raising awareness of mental errors (i.e., EF problems) and attentional slips that hinder participants from acting in a goal-oriented and efficient manner (Stamenova and Levine, 2019). Participants learn how to self-monitor and redirect attention with the use of mindfulness techniques. Moreover, GMT has a primary focus on daily life EFs. For instance, participants are encouraged to stop and monitor their own behavior in daily life relative to current goals. GMT and GMT principles have been found to improve both neurocognitive performance and self-reported EFs for diverse conditions, such as ABI [e.g., Levine et al. (2000) and Tornås et al. (2016)], age-related cognitive decline (Levine et al., 2007), schizophrenia (Levaux et al., 2012), spina bifida (Stubberud et al., 2013, 2014), and attention deficit hyperactivity disorder (ADHD) (Jensen et al., 2021). Current results also suggest that GMT has the capacity to improve aspects of mental health (Levaux et al., 2012; Stubberud et al., 2015; Boyd et al., 2019; Hagen et al., 2020) and HRQoL (Cuberos-Urbano et al., 2018).

As GMT precisely targets the cognitive difficulties that adult ALL survivors experience (i.e., EFs and attention), there is reason to believe that they could benefit from this type of cognitive rehabilitation. Furthermore, the real-life focus of GMT would be valuable to ALL survivors who struggle in various domains of life (Kanellopoulos et al., 2013; Krull et al., 2013b). Thus, a future clinical trial is warranted. However, the feasibility and acceptability of GMT in adult ALL survivors are unknown. Such knowledge is crucial for the planning of a successful future randomized controlled trial (RCT). Furthermore, the rehabilitation potential of neurocognitive late effects in this group is unexplored.

The first aim of this study was to explore the feasibility and acceptability of a group-based GMT for adult ALL survivors to use in a future multicenter RCT. To examine feasibility, we evaluated adherence (e.g., attendance, completion of home exercises), feedback on program structure (participants and therapists), and safety (i.e., adverse effects). In addition, acceptability was examined by questions addressed to participants concerning usefulness, suitability, and satisfaction with the program. The second aim of the study was to explore whether GMT was associated with reliable changes. To achieve this, a case-series design was employed, in which self-reported EFs and neurocognitive outcomes were evaluated preintervention, postintervention and at 6 months follow-up across individual cases of adult ALL survivors with EF complaints.

## Materials and Methods

### Participants and Procedures

Study invitation letters were distributed to eight childhood ALL survivors via a cancer late effects clinic at St. Olavs Hospital, Trondheim University Hospital, Norway, and social media. Four subjects contacted via the late-effects clinic, and one subject contacted via social media replied. One replier eventually declined due to the time required and because she had minimal EF complaints. Thus, the final sample consisted of four participants who all met the eligibility criteria of being survivors of childhood ALL with a minimum of 5 years post diagnosis and between 18 and 40 years of age (see Table 1 for demographic characteristics of the sample). Two of the participants reported to have reduced occupational capacity because of neurocognitive late effects and fatigue, while the remaining participants did not report such limitations. Furthermore, while all participants reported EF complaints, all had an average or above average estimated general ability level (GAI IQ score range 91–117) at baseline. None of the participants had a history of premorbid central nervous system injury or disease or premorbid ADHD. Furthermore, none of the participants had previously received cognitive rehabilitation or were receiving other cognitive rehabilitation during study participation.

**TABLE 1:** Demographic characteristics of the sample at baseline (*n* = 4).

	Median (Range)
Years of age	31.5 (28–39)
Years of education	14 (12–14)
Age at diagnosis, years	4.5 (2–13)
Time since diagnosis, years	26 (23–32)
Time since last cancer treatment, years	24 (16–30)

*Numbers in parentheses represent the range.*

All baseline measures were collected during September/October 2019, postintervention measures collected during February 2020 and 6-month follow-up measures during August/September 2020. All self-report measures were employed at baseline, 14 days postintervention and 6 months after completing GMT (6-month follow-up). This did not include assessments of feasibility and acceptability, as these were only conducted at follow-up. Similarly, neurocognitive functioning was assessed at baseline, postintervention (14 days) and at the 6-month follow-up, except for the WAIS-IV, which was administered at baseline only. The administration of neurocognitive tests was performed by a technician who was not involved with the delivery of GMT.

### Intervention

Goal Management Training is a manualized cognitive rehabilitation program with an original English protocol (Levine et al., 2011). The present study used the Norwegian translation of this protocol (Stubberud et al., 2013; Tornås et al., 2016) consisting of nine modules with accompanying PowerPoint slides, participant workbooks and a wide range of EF (e.g., multitasking) and attention (e.g., mindfulness) (Kabat-Zinn and Hanh, 2009) exercises (Table 2). The participants received the modules in a group of four for a total of five sessions over a period of 3 months (November 2019–January 2020). All sessions were led by a licensed psychologist (author TR) and a co-therapist (author KE). Except for session 1, which contained only module 1, participants received two modules in each session (see Table 2). The overall focus was to increase participants’ attentional and problem-solving capacity through the learning of compensatory strategies (e.g., to stop the autopilot and to monitor behavior regarding one’s current goals) to improve real-life EFs. Home exercises consisted of monitoring episodes of inattention and performing mindfulness exercises in addition to applying learned strategies in daily life. All participants received external cuing by text messages reading “STOP!” following session 3 and throughout the intervention period at random daytime hours (12 per participant). In addition, group discussions of daily life EFs were a central part of the rehabilitation. Each session lasted approximately 5 h, including a 1-h lunch break. Two modules were combined in each session to reduce traveling for participants who lived far away.

**TABLE 2:** Session structure of GMT modules.

Sessions and GMT modules	Key concepts and objectives
**Session 1**	
Module 1 Present mindedness absentmindedness	1 Introduction of goal hierarchies, the “mental workshop,” absentmindedness and present mindedness
**Session 2**	
Module 2 Absentminded errors	2 Increase awareness of how absentmindedness can lead to errors
Module 3 The autopilot	3 Introduction of the autopilot: advantages and disadvantages of the autopilot
**Session 3**	
Module 4 Stop the autopilot	4 Learn how to stop the autopilot and monitor one’s own behavior
Module 5 The mental blackboard (Working memory)	5 Learn how to stop and to check one’s mental blackboard (Working memory)
**Session 4**	
Module 6 State your goal	6 State your goal: remembering it and completing it
Module 7 Decision-making	7 Goal conflicts, complex tasks and stress: Stop! (breathe- be present), state your goal
**Session 5**	
Module 8 Splitting tasks into subtasks	8 When the mental blackboard is too small: Splitting complex tasks into subtasks and concretizing them (to-do list)
Module 9 Checking (Stop!)	9 Summary of GMT modules and elaboration of the Stop- State-Split- Check technique

### Evaluations

#### Feasibility

Session attendance (%), completion of home exercises, use of strategies from GMT at the 6-month follow-up, and outcome measure completion were explored to characterize adherence to study procedures and cognitive rehabilitation. A questionnaire previously used in Stubberud et al. (2020) was used to measure adherence. Participants were asked whether they still used strategies from the program and to what degree (1 = *very rarely* to 5 = *very often*). Two additional items were added for this study to assess adherence (*How many of the home exercises did you complete?* and *How often did you practice home exercises?*). Items were scaled from 1 (*almost none/very rarely)* to 5 (*almost all/very often*). Finally, the number of participants completing outcome assessments at the 2-week and 6-month follow-ups was examined. A custom-made interview was designed to provide feedback regarding GMT procedures and protocols. Participants were asked about practical aspects of the program, including group size, number of sessions, session time length, number of breaks and positive aspects of the program, aspects that could be improved or whether they had any other thoughts on the program. In addition, the therapist and co-therapist evaluated the implementation of the GMT protocol. Finally, potential adverse events related to GMT or study procedures were registered.

#### Acceptability

The same questionnaire used to assess adherence (Stubberud et al., 2020) also included seven items relating to the acceptability of GMT. Specifically, questions were designed to assess (1) usefulness (1 = *not useful* to 5 = *very useful*), (2) suitability (1 = *not suitable* to 5 = *very suitable*), and (3) satisfaction by items asking whether participants would be willing to participate if a similar study were offered to them (*yes/no*) and whether they would recommend rehabilitation to others in the same situation (*yes/no*).

#### A Semistructured Interview

A semistructured interview was used to collect demographics (e.g., *what is your education level today*?), psychological (e.g., *negative life events within the last 12 months*), and medical information (e.g., *have you ever had a traumatic brain injury*?). In addition, EF complaints were assessed in this interview at baseline to assess eligibility.

#### Self-Report Measures

The Behavior Rating Inventory of Executive Function, Adult [BRIEF-A, Roth et al. (2005)] was used to assess daily life EFs. The questionnaire provides a Global Executive Composite (GEC) score and two index scores: the Behavioral Regulation Index (BRI) and the Metacognitive Index (MI). The questionnaire also provides the more specific subscales of Inhibit, Shift, Emotional control, Self-Monitor, Initiate, Working memory (WM), Plan/Organize, Task Monitor and Organization of Materials. From these scales, we considered Shift, WM, Plan/Organize and Task Monitor most relevant regarding the focus of GMT. Raw scores were converted to *T* scores (*M* = 50, *SD* = 10), where higher scores indicate worse EF. Test-retest reliability *r* = 0.82–0.94 (Roth et al., 2005).

Additional self-report measures were employed to examine the psychosocial health of the group. From the Quality of Life Inventory (PedsQL^TM^4.0) young adult version (Varni and Limbers, 2009), a total summary health score (23 items) was computed. Items were reverse scored and transformed to a scale range of 0–100, of which higher scores indicate higher HRQoL. In a Dutch study, the total score of healthy young adults was *M* = 85.88 (*SD* = 11.45) and *M* = 76.65 (*SD* = 15.92) in young adults with chronic health conditions (Limperg et al., 2014), demonstrating adequate construct validity in European adults.

The Hopkins symptom checklist (HSCL-25) (Derogatis et al., 1974) measured adult mental health symptoms within the previous week, with 25 items ranging from 1 = *not at all* to 4 = *extremely*. A mean item score was calculated, in which ≥1.75 is a recommended clinical cutoff point (Strand et al., 2003).

To assess fatigue, we used the Fatigue Severity Scale (FSS) (Krupp et al., 1989), where symptom severity is rated on a seven-point Likert scale (*1 = strongly disagree to 7 = strongly agree)* (Krupp et al., 1989). Based on previous reports on the prevalence of fatigue, a mean FSS score of ≥5 was interpreted as indicative of severe fatigue (Lerdal et al., 2005).

#### Neurocognitive Test Battery

The neurocognitive outcome measures consisted of a wide range of neurocognitive tests intended to characterize cognitive functioning. Block design, matrix reasoning, similarities, vocabulary and digit span (Scaled scores, *M* = 10, *SD* = 3) from the Wechsler Adult Intelligence Scale-IV (WAIS-IV; Wechsler (2008)) was used to calculate the general ability index (GAI, IQ scores, *M* = 100, *SD* = 15) as a measure of intellectual functioning at baseline. Higher scores reflect better performance (Wechsler, 2008).

Furthermore, the California Verbal Learning Test (CVLT II) *(Verbal memory)* was used to assess verbal learning, memory, and attention span. Raw scores were converted to *Z*-scores (*M* = 0, *SD* = 1) and *T*-scores (*M* = 50, *SD* = 10), where higher scores reflect better performance except for error measures. Test-retest reliability ranges from *r* = 0.27 to *r* = 0.88 with a test interval of nine to 49 days. For Trial 1 correct recall, a measure of auditory attention span, a reliability of *r* = 0.57 has been demonstrated (Delis et al., 2000). The alternative version was used at the 14-day follow-up. The remaining subset of tests was used to measure attention, EF and processing speed: Conner’s Continuous Performance Test 3rd Edition (CPT-3) (Conners, 2014) was used to assess attention and inhibition (*M* = 50, *SD* = 10), where higher T-scores for this study reflect better performance (reverse scored). The CPT-3 scores demonstrate strong reliability and stability across repeated administrations (seven to 35 days), with a median test-retest *r* = 0.67 and *r* = 0.85 for Commissions.

The Wisconsin Card Sorting Test (WCST-4) (Heaton et al., 1993, 2004) was used to assess problem solving capacity, cognitive set-shifting and abstraction (Lezak et al., 2012). A computer-based version was employed where raw scores and *T*-scores (*M* = 50, *SD* = 10) were generated. Perseverative responses and perseverative errors are sensitive to cognitive set shifting (Lezak et al., 2012). For raw scores, higher scores reflect worse performance, while for T-scores, higher scores reflect improved performance. These measures have been found to show strong test-retest stability in healthy adults (*r*_*I*_ = 0.68 and *r*_*I*_ = 0.72) with a 9-month test retest interval (Tate et al., 1998).

The Color-Word Interference test (CWI) from the Delis–Kaplan Executive Function System is a measure of inhibition and cognitive set-shifting (Delis et al., 2001). The test consists of four conditions: color naming (condition 1), word reading (condition 2), inhibition (condition 3) and inhibition switching (condition 4). Conditions 1 and 2 were used to assess processing speed in the current study. Primary scores are computed for completion time (condition 1–4). Raw scores are converted to scaled scores (*M* = 10, *SD* = 3), where higher scores reflect better performance. Test-retest reliability for primary measures was in the moderate to high range with *r* = 0.49–0.86 for ages 20–49.

The Trail Making Test (TMT) from the *Delis–Kaplan Executive Function System* (Delis et al., 2001) is a measure of cognitive set-shifting. The primary measure is condition 4, where the participant is asked to shift between number and letter sequencing. The test also measures the basic abilities of visual scanning (condition 1), motor pace (condition 5) and number- and letter sequencing combined with drawing a line (condition 2 and 3). The latter two were used to assess processing speed. Scores reflect completion time, and raw scores are converted to scaled scores (*M* = 10, *SD* = 3), where higher scores reflect better performance. Test retest reliability was in the moderate to high range, *r* = 0.48–0.73 for ages 20–49.

Finally, the hotel task (Manly et al., 2002) was used to examine EF in a real-life multitasking situation (Shallice and Burgess, 1991) where the structure from the test technician is reduced to a minimum. The participant is instructed to play the role of a hotel manager with five tasks. Within a time window of 15 min, the participant is asked to spend as much time as possible on each task (i.e., distribute time equally between the tasks). As such, the task is designed to assess higher-level EF (planning, organization, and self-monitoring) in “real life”. Raw scores are computed for the number of “tasks attempted” and “deviation from optimal time use.” Higher scores reflect better performance in “tasks attempted” (max. of five) but worse performance in “deviation of optimal time use” (seconds above or below the optimal 3 min per task). Previous studies suggest that test results may reveal EF impairment not captured by traditional assessment (Torralva et al., 2013).

### Statistical Analysis

Descriptive statistics were analyzed with IBM SPSS Statistics 25. The Reliable Change Index [RCI, Jacobson and Truax (1991)] was calculated in Excel to assess whether changes in EF scores, processing speed and attention represented a statistically reliable change from preintervention to the 6-month follow-up using the following formula:


$$R\,C\,I=\left(X^{2}-X^{1}\right)/S\,E_{d\,i\,f\,f}$$

Individual scores from baseline were subtracted from individual 6-month follow-up scores (X^2^–X^1^) divided by the standard error of the difference (SE *_*diff*_*), which was derived from the following formula:


$$S\,E_{d\,i\,f\,f}=\sqrt{2\,{\left(S\,E\right)}^{2}}$$

Furthermore, the standard error (S_*E*_) was derived from the standard deviation (S) of the score and the test-retest reliability of the measure (r_*xx*_):


$$S_{E}=S\,\sqrt{\left(1-r_{x\,x}\right)}$$

An RCI value exceeding the threshold of 1.96 (either RCI > 1.96 or RCI < −1.96) is unlikely to reflect fluctuations of an imprecise measurement (*p* < 0.05) (Jacobson and Truax, 1991). Thus, the RCI value indicates the reliability of the score change from baseline to follow-up. Due to the exploratory nature of this study, RCI was calculated for BRIEF-A (subscales, indices, and GEC) and neurocognitive test performance on tests where at least one participant demonstrated impairment at baseline (*1.5 SD below the normative mean*). Reliability estimates (see outcome measures) and standard deviations needed for computations were taken from the manuals (Delis et al., 2000, 2001; Roth et al., 2005; Conners, 2014) and Tate et al. (1998). Score changes were computed from raw scores (BRIEF-A, CVLT-II, and WCST-4) or standardized scores (CPT-3, TMT, and CWI) associated with age at baseline only to reduce the influence of changing norm group from baseline to follow-up.

### Compliance With Ethical Standards

The study was approved by the Regional Committee for Medical Research Ethics in Central Norway (2018/1810/REK Midt) and was conducted in accordance with the Declaration of Helsinki. All participants provided written informed consent prior to study participation.

## Results

### Descriptive Statistics for Self-Report Measures and Neuropsychological Scores at Baseline

Descriptive statistics of self-reported psychosocial health can be found in Table 3. All four participants scored above the clinical cutoff ≥5 for the FSS score, indicating severe fatigue. In addition, two of the participants scored above the clinical cutoff (1.75) for the HSCL, and the HRQoL total score was low compared to that of healthy young adult samples (Varni and Limbers, 2009; Limperg et al., 2014). For self-reported EF and neurocognitive test scores at baseline, see Tables 4, 5.

**TABLE 3:** Descriptive statistics of self-reported psychosocial health.

Mean (SD)	Participant 1	Participant 2	Participant 3	Participant 4
**FSS**				
5.61 (0.69)	5.67	5.00	6.56	5.22
**HSCL**				
1.93 (0.83)	1.40	1.12	2.96	2.24
**PEDSQL**				
60.60 (1.86)	60.87	63.04	58.70	59.78

*FSS, Fatigue Severity Scale; HSCL, Hopkins Symptom Check List; PEDSQL, Pediatric Quality of Life Inventory. Clinical cutoff for FSS *M* = ≥ 5 and for HSCL *M* = 1.75. *M* = 85.88 (*SD* = 11.45) in the PEDSQL total score for healthy young adults (Limperg et al., 2014).*

**TABLE 4:** Reliable change index for self-reported executive function in daily life (BRIEF-A).

Variable	Participant 1			Participant 2			Participant 3			Participant 4		
	Pre	F/U	RCI	Pre	F/U	RCI	Pre	F/U	RCI	Pre	F/U	RCI
Shift	74	61	**−2.88**	64	74	1.85	74	74	0	73	73	0
Emotional	53	54	0	40	53	**2.39**	81	71	−1.88	60	67	1.44
Initiate	54	52	−0.73	56	57	–1.14	61	54	−1.45	66	85	**3.43**
WM	91	83	**−2.76**	76	81	0.81	67	53	**−3.68**	69	79	**2.42**
Plan/org	55	51	−1.01	65	66	0	47	47	0	65	68	0.45
BRI	52	51	−0.55	46	56	**2.31**	73	64	**−2.20**	62	68	1.54
MI	63	60	−1.11	65	67	–0.41	53	47	−1.78	65	79	**4.01**
GEC	59	57	−1.03	58	63	1.09	62	55	**−2.36**	65	76	**3.26**

*Scores are reported as *T*-scores (*M* = 50, *SD* = 10), and higher scores reflect increased symptoms. RCI derived from raw scores and negative RCI values indicate reduced symptoms, >1.96 are significant at *p* < 0.05 and highlighted with a bolded format. Pre, baseline scores; F/U, 6-month follow-up; RCI, Reliable Change Index.*

**TABLE 5:** Reliable change index for neurocognitive function.

Variable	Participant 1			Participant 2			Participant 3			Participant 4		
	Pre	F/U	RCI	Pre	F/U	RCI	Pre	F/U	RCI	Pre	F/U	RCI
**CPT-3**												
Commission	41	46	1.01	44	46	0.41	**35**	**46**	**2.23**	46	51	1.01
**CVLT-II**												
Imm. Recall trial 1	5	10	1.91	6	7	0.38	5	6	0.38	7	8	0.38
**WCST-4**												
Pers. response	**40**	**5**	**−2.53**	4	4	0	41	30	−0.80	14	8	−0.43
Pers. error	**33**	**5**	**−2.44**	4	4	0	32	27	−0.44	14	8	−0.52
**TMT**												
Cond. 3	12	12	0	**5**	**12**	**2.07**	11	9	−0.59	8	11	0.89
**CWI**												
Cond. 2	6	6	0	3	5	0.68	10	13	1.01	6	8	0.68

*Standardized *T*-scores (*M* = 50, *SD* = 10) associated with age at baseline are reported for CPT-3, raw scores are reported for CVLT-II and WCST-4, and scaled scores (*M* = 10, *SD* = 3) associated with age at baseline are reported for D-KEFS variables. Positive RCI values indicate improved performance for CPT-3, CVLT-II, and D-KEFS. For WCST-4, negative RCI values indicate improved performance (due to measurement direction). RCI < 1.96 are significant at *p* < 0.05 and highlighted with a bolded format. Pre, baseline scores; F/U, 6-month follow-up; RCI, Reliable Change Index.*

### Feasibility and Acceptability

#### Feasibility

Two of the participants attended all five group sessions (100% attendance), while the other two participants missed one group session each (Sessions 3 and 4, respectively) due to personal reasons (80% attendance). Completion of home exercises varied somewhat within the group. One participant completed “almost all” exercises, another completed “almost none,” and the remaining two participants reported having completed “some” and “about half” of the exercises. All participants reported that they were still using strategies from the program at the 6-month follow-up, although the degree to which they did this varied. Participant 1 reported using the strategies *often* (4), participants 3 and 4 reported using the strategies *sometimes* (3), and participant 2 reported using them *rarely* (2). Outcome measures at the 2-week and 6-month follow-ups were completed by all participants. Regarding program structure, the group size of four was considered appropriate among all participants, and all participants confirmed that they were satisfied with the five-session structure. Being part of a group was mentioned as the most positive aspect of the intervention by all participants (e.g., exchanging experiences, meeting other childhood cancer survivors, and group discussions). Mindfulness exercises were mentioned by two participants (participants 4 and 1), while the “STOP!” exercise/techniques were mentioned by three participants (participants 1, 2, and 3) as being the most useful. Regarding improvement potential, two participants requested more frequent breaks to avoid sleepiness and mental overload, and two participants also recommended fewer home exercises. Similarly, evaluation from the group therapists concluded that the group size and number of sessions were feasible. However, the session time schedule was somewhat tight, which reduced the time available for discussions. Furthermore, combining two modules in each session meant an increased number of home exercises between sessions. As a result, it was suggested that the number of home exercises be somewhat reduced. However, to increase adherence regarding home exercises, it was also suggested that the importance of completing the exercises be more strongly emphasized.

Regarding safety, no adverse events were registered.

#### Acceptability

All participants rated the intervention as useful and suitable. Two of the participants rated it (5) *very useful* and (5) *very suitable*, while the other two rated it (4) *useful* and (4) *suitable*. Furthermore, all participants reported being willing to participate if a similar study were offered to them and would recommend the intervention to others in the same situation.

### Semistructured Interview

Negative life events were registered for three of the participants (participant 1, 3, and 4) during the study participation period. These life events were unrelated to study procedures and protocol.

### Change in Self-Reported Executive Function and Neurocognitive Test Performance

The results from the analysis of BRIEF-A scores show that participants 1 and 3 obtained a negative RCI below −1.96, which indicated a reliable improvement in daily life EF (Table 4). Participant 1 showed improvements in Shift and WM, while participant 3 showed improvements in WM, BRI and GEC. In contrast, participants 2 and 4 obtained a positive RCI above 1.96 on the BRIEF-A, indicating a reliable self-reported deterioration in daily life EFs (Table 4). Participant 2 showed worse emotional control and BRI, while participant 4 showed worse daily life EF in Initiate, WM, MI, and GEC. Participants 1, 2, and 3 obtained an RCI exceeding ±1.96 at the neurocognitive test follow-up, indicating improved performance on the CPT-3, WCST-4, and TMT (Table 5).

### Change in Real Life Executive Function and Self-Reported Psychosocial Health

**FIGURE 1:**
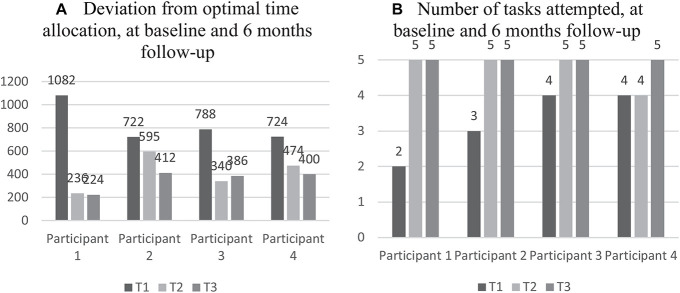
Individual scores from the hotel task at preintervention, postintervention, and 6-month follow-up. **(A)** Raw scores of “deviations from optimal time allocation” are presented in seconds. Higher scores indicate worse performance. **(B)** Number of tasks attempted are reported in raw scores. Higher scores indicate better performance.

**FIGURE 2:**
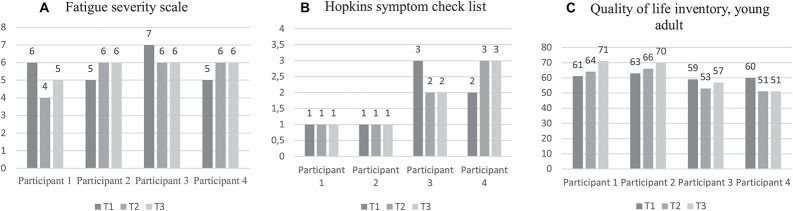
Self-reported psychosocial health at preintervention, postintervention, and 6-month follow-up. **(A)** Mean scores as measured by Fatigue severity scale (FSS) are presented. Higher scores indicate higher fatigue. **(B)** Mean scores as measured by Hopkins’s Symptom Check List (HSCL-25) are presented. Higher scores indicate a higher symptom level. **(C)** Total summary health scores, as measured by the Quality of life inventory (PedsQL^TM^4.0), are presented. Higher scores indicate higher health-related quality of life (HRQoL).

As illustrated in Figures 1A,B, there was considerable improvement in the hotel task in “optimal time allocation” and “number of tasks attempted” changes in self-reported psychosocial health is shown in Figures 2A–C.

## Discussion

While GMT is a promising program for the rehabilitation of neurocognitive late effects, information regarding the feasibility of GMT for adult ALL survivors is lacking. The first aim of the present study was therefore to examine the feasibility and acceptability of a group-based GMT program for adult ALL survivors. The results showed that adherence to GMT and study-related procedures was strong. Although home exercise completion varied, attendance of sessions was good (80–100%), and all outcome assessments were completed.

Compared to earlier studies (Van Hooren et al., 2007; Alfonso et al., 2011; Levine et al., 2011; Boyd et al., 2019), the number of sessions in the current study was fewer (five sessions) and sessions were stretched out over a longer time period (3 months). This session structure (i.e., five group sessions) was employed to reduce traveling in this geographically dispersed population. Our results suggest that all participants and the two therapists were satisfied with this structure. Still, adherence was not ideal. Two of the participants were not able to attend in all sessions (80% attendance). For a future RCT study, digital delivery of GMT sessions should be considered as an alternative solution for participants who have time limitations. Furthermore, this could also ensure adherence in other situations where participants are unable to attend physically (e.g., due to the coronavirus disease). Also, a subset of participants completed fewer home exercises than expected. Thus, some participants did not adhere to the protocol for home exercises, despite EF complaints. Indeed, home exercises are a central part of GMT, and intended to facilitate EF in real life. The importance of home exercises for improved daily life EF should therefore be more strongly emphasized to participants in a future RCT.

Furthermore, being part of a group was reported as a positive aspect of GMT by all participants. Thus, although GMT can be delivered individually (e.g., in person or digitally), the group format may be a motivating factor for adult ALL survivors. Participants also appreciated the central STOP technique (“stop and think”) and mindfulness training, and the results did not indicate any need for changes (adaptations or additional materials) to be made to the protocol.

In fact, the intervention was very well received among the participants. All four participants rated the rehabilitation as useful, suitable, and satisfactory. This is a promising finding, indicating that this type of cognitive rehabilitation is pertinent to address the needs of this population. Finally, no adverse events were registered, suggesting that the intervention was well tolerated, and that GMT is both acceptable and safe for adult ALL survivors.

To our knowledge, there are no studies of cognitive rehabilitation in adult ALL survivors. Thus, the second aim of this study was to explore whether GMT was associated with reliable changes in long-term neurocognitive late effects. Although exploratory, it is interesting to note that the neurocognitive test results showed improved performance in three of the participants. This is consistent with the objective of GMT (Levine et al., 2011) and with research in other populations following GMT (Levine et al., 2011; Stubberud et al., 2013; Tornås et al., 2016). In contrast, participant 4 did not show reliably improved neurocognitive function.

The primary objective of GMT, however, is to improve daily life EF. Notably, the results indicated improvement in daily life EF of participants 1 and 3 in the areas of shifting, WM, BRI, and GEC. While WM is closely related to attention, shifting involves switching or alternating attention/focus, both of which are expected to improve following GMT (Levine et al., 2011). Moreover, these are the same functions required by our performance measures of attention (CPT-3) and cognitive set-shifting (WCST-4). Thus, these two participants improved both *performance* and daily life functioning in these domains. Moreover, they both showed improvements in self-reported psychosocial health post intervention and at 6-month follow-up. Importantly, participants 1 and 3 reported using the GMT at the 6-month follow-up. This indicates that these participants had internalized compensatory strategies and applied them in daily life following GMT.

Of note, the remaining two participants did not show improved daily life EF. Several factors such as the severity of neurocognitive late effects, may be associated with the outcome of rehabilitation. It is notable that both participant 2 and 4 scored above the clinical cutoff (*T* ≥ 65) on the MI (BRIEF-A, Table 4), suggesting problems with metacognition. As the GMT protocol involves high metacognitive demands (Stamenova and Levine, 2019), these particular problems could have made the program especially challenging. Further, it does not seem likely that general abilities can explain the poor outcomes in participant 2 and 4, as all participants in the current study displayed abilities within the average range (GAI > 85). However, it should be noted that participant 4 demonstrated below average verbal ability at baseline (WAIS-IV, Vocabulary, *S* = 7). Since the GMT is also a verbally demanding intervention, low verbal ability may have an impact on responsiveness. Thus, it is possible that a subset of adult survivors of childhood ALL may not profit optimally from this type of rehabilitation program. In future research, the role of preintervention functioning (i.e., ability, occupational status) should therefore receive further attention.

Another interpretation is that the increased awareness that may result from GMT, can also cause an increase in EF complaints, and so cause a worsening in some scores on the BRIEF-A. However, improvement might still be evident in other areas of daily life functioning. Indeed, HRQoL did show some improvement in participant 2.

In contrast, self-reported psychosocial health showed a marked deterioration in participant 4, which further supports the interpretation of reduced responsiveness in this subject. Several factors unrelated to GMT, such as the registered negative life events and the COVID-19 pandemic may have contributed to these outcomes (Blix et al., 2021; Ebrahimi et al., 2021). Moreover, since the BRIEF-A has been found to be strongly associated with psychological distress (Løvstad et al., 2016), it is possible that psychological distress also affected the increased EF complaints of participant 4 at follow-up. Thus, for several reasons, a more objective outcome measure of daily life EF such as the goal attainment scale (GAS, Kiresuk and Sherman, 1968; Bovend’Eerdt et al., 2009) or informant reports (e.g., spouse) should be considered in future research.

Also relevant to the concept of generalization, however, is the hotel task, which mimics a real-life situation with high demands on EF (i.e., multitasking). In line with earlier studies (Richard, 2013; Richard et al., 2019), results showed reduced “deviation from optimal time use” and an increase in “number of tasks attempted.” Interestingly improvement in this task was found in all participants following GMT, both postintervention and at the 6-month follow-up. This may indicate improved higher-level EF, including planning and organization, and an increased level of self-monitoring. Although practice effects cannot be ruled out, it is not unrealistic that the intensive training of goal processing can account for the overall improvement in this task. Especially since the main pitfall of this task is to forget the overarching goal, namely, to distribute time equally between all tasks. Moreover, it has previously been demonstrated that performance on the hotel task was significantly associated with attentional control, supporting the training of attentional control to improve EF performance in real-life settings (Stubberud et al., 2013).

Finally, it should be noted that there is currently little knowledge about the level of insight in ALL survivors. Participants in future research may show reduced insight, especially, since knowledge of neurocognitive late effects may be limited (Ruud et al., 2012; Lee et al., 2019). Thus, in a future RCT, neurocognitive impairments (not only complaints) may be considered as part of inclusion.

## Limitation

There are several limitations in the current study. First, conclusions regarding feasibility and acceptability should be interpreted with caution due to the small sample size. Several factors (e.g., over/underreporting of symptoms, awareness, demand characteristics, cognitive impairments, or social desirability bias) may also affect the accuracy and validity of self-reports. Further, due to the exploratory nature of this study, we analyzed change in areas where participants displayed impairments. This could pose a problem with regression to the mean (i.e., natural variation in repeated data). Therefore, a design with multiple data points at baseline (self-reports) would have been ideal to provide experimental control. However, due to the extensive neurocognitive testing involved in this study, repeated measures at baseline were not performed. To reduce this problem in future research, specific hypothesis should be stated at the outset of the study. Another limitation with the neurocognitive test results in this study is practice effects, which may have contributed to the reliable change that was found. Neurocognitive tests of EFs such as the WCST and the hotel task may be particularly susceptible to practice effects. Still, improved performance in these tests might be expected following GMT due to several in session exercises inherent in the GMT program. These include exercises focused on multitasking, shifting and stopping the autopilot. Moreover, the practice effects for CPT-3 variables have previously been described as small (−2.9 *T*-score points for Commission) and have been found to deteriorate rather than to improve (Conners, 2014). Furthermore, it has been reported in several meta-analyses that practice effects are most pronounced between the first and second administration of a test, with smaller increases for subsequent administrations (Scharfen et al., 2018a,b). The fact that improvements were still present in the hotel task at 6-month follow-up suggest that at least some of the improved performance in real-life EF could be associated with internalized compensatory strategies acquired in GMT. Nonetheless, a future RCT is invaluable for examining the effect of an intervention and will reduce the influence of the methodological problems stated above.

## Conclusion

This is the first study to explore the feasibility of GMT for adult survivors of childhood ALL. Preliminary results suggest that the GMT protocol is feasible and acceptable for ALL survivors, despite high levels of fatigue and relatively low HRQoL. Furthermore, our results also suggest that GMT may have the potential to produce reliable improvements in daily life EF and neurocognitive functioning for adult ALL survivors with long-term late effects. Notably, these findings may inform clinicians working with patients exhibiting long-term ALL late effects, as well as researchers planning to evaluate cognitive rehabilitation in ALL survivors. However, whether GMT may lead to the above-mentioned improvements in this population remains to be examined in a future RCT.

## Data Availability Statement

The datasets presented in this article are not readily available because due to the nature of this research, participants of this study did not agree for their data to be shared publicly, so supporting data is not available. Requests to access the datasets should be directed to KE, Kaja.solland.egset@ntnu.no.

## Ethics Statement

The studies involving human participants were reviewed and approved by the Regional Committee for Medical Research Ethics in Central Norway (REK Midt). The patients/participants provided their written informed consent to participate in this study.

## Author Contributions

KE had the primary role of writing the manuscript and performing analyses. JS and OH helped with statistical analyses. JS contributed with expert knowledge of GMT. SW contributed with planning and supervision of neurocognitive testing procedures. MH contributed with medical data extraction. MR contributed with her experience with the hotel task. TR, SW, JS, OH, ER, MH, M-EE, AS, KE, and MR contributed to the conception and design of the study. TR supervised the study from beginning to end. All authors contributed with valuable inputs to manuscript revision, read, and approved the submitted version.

## Conflict of Interest

The authors declare that the research was conducted in the absence of any commercial or financial relationships that could be construed as a potential conflict of interest.

## Publisher’s Note

All claims expressed in this article are solely those of the authors and do not necessarily represent those of their affiliated organizations, or those of the publisher, the editors and the reviewers. Any product that may be evaluated in this article, or claim that may be made by its manufacturer, is not guaranteed or endorsed by the publisher.
